# Transcatheter Closure of Atrial Septal Defect: A Review of Currently Used Devices

**DOI:** 10.7759/cureus.40132

**Published:** 2023-06-08

**Authors:** Shitij Shrivastava, Shashwat Shrivastava, Sai Vishnu Vardhan Allu, Patrik Schmidt

**Affiliations:** 1 Internal Medicine, BronxCare Health System, New York, USA; 2 Cardiothoracic Surgery, New York University, New York, USA

**Keywords:** patent foramen ovale, interventional cardiology, percutaneous atrial septal defects closure, cogenital heart disease, gore cardioform septal occluder, amplatzer cribriform occluder, amplatzer septal occluder, percutaneous pfo closure, transcatheter closure device, ostium secundum atrial septal defect

## Abstract

Over the past seven decades, significant advancements and innovations have occurred in the field of percutaneous atrial septal defect (ASD) closure using transcatheter-based devices. This article focuses on the current literature surrounding the three Food and Drug Administration (FDA)-approved devices for ASD and patent foramen ovale (PFO) closure in the United States, namely, the Amplatzer Septal Occluder (ASO), Amplatzer Cribriform Occluder, and Gore Cardioform ASD Occluder. The ASO has been widely used since its FDA approval in 2001. Studies have shown its high success rate in closing ASDs, especially small-sized defects. The RESPECT trial demonstrated that PFO closure using the ASO reduced the risk of recurrent ischemic stroke compared to medical therapy alone. The Closure of Atrial Septal Defects With the Amplatzer Septal Occluder Post-Approval Study (ASD PMS II) evaluated the safety and effectiveness of ASO in a large cohort of patients, reporting a high closure success rate and rare hemodynamic compromise. The Amplatzer Cribriform Occluder is designed for the closure of multifenestrated ASDs and has shown promising results in small-scale studies. It successfully closed the majority of fenestrated ASDs, leading to improved right ventricular diastolic pressure without major complications. The REDUCE trial compared PFO closure using the Gore Helex Septal Occluder and Gore Cardioform Septal Occluder with antiplatelet therapy alone. The study demonstrated that PFO closure significantly reduced the risk of recurrent stroke and brain infarction compared to antiplatelet therapy alone. However, the closure group had a higher incidence of atrial fibrillation or atrial flutter. There is a risk of atrial fibrillation with the use of ASO as well. The FDA-approved Gore Cardioform ASD Occluder showed excellent performance in the ASSURED clinical study. The device achieved high technical success and closure rates, with low rates of serious adverse events and device-related complications. A meta-analysis comparing transcatheter ASD closure with surgical closure revealed that the transcatheter approach had a high success rate, lower rates of adverse events, and shorter hospital stays compared to surgery, without any mortality. Complications associated with transcatheter ASD closure have been reported, including femoral arteriovenous fistulas, device embolization, cardiac erosion, aortic incompetence, and new-onset migraine. However, these complications are relatively rare. In conclusion, transcatheter ASD closure using FDA-approved devices has proven to be safe and effective in the majority of cases. These devices offer excellent closure rates, reduced risk of recurrent stroke, and shorter hospital stays compared to surgery. However, careful patient selection and follow-up are necessary to minimize complications and ensure optimal outcomes.

## Introduction and background

The interatrial septum is a structure that separates the right atrium from the left atrium. The development of the interatrial septum begins during the fifth week of gestation. An atrial septal defect (ASD) may arise when there is a defect in the formation of the interatrial septum which leads to communication between the right and left atrium. There are essentially four types of atrial septal defects, namely, ostium primum ASD, ostium secundum ASD, sinus venosus ASD, and unroofed coronary sinus ASD. Sinus venosus ASD and unroofed coronary sinus ASD are not defects of the interatrial septum but they behave like defects of the interatrial septum as they allow communication between the right and left atrium [1]. ASDs are the most common type of congenital heart defects to present in adulthood [2]. Common atrium is a rare pathology observed when the septum primum, septum secundum, and superior and inferior endocardial cushions fail to develop. A patent foramen ovale (PFO) is a subtype of ostium secundum defect and occurs when the septum primum and septum second fail to close once the infant begins breathing [3]. Transcatheter device closure of ASD is the preferred way of closure of septum secundum defects [4]. The first transvenous device used to close an ASD was utilized by King and Mills in 1974. They used an experimental device in 13 animals who had an ASD punched experimentally. Five animals achieved successful closure [5,6]. As of now, three devices are Food and Drug Administration (FDA) approved for ASD closure in the United States, namely, Amplatzer Septal Occluder (ASO), Amplatzer Cribriform, and Gore Cardioform Septal Occluder. This article will focus on the current literature on the above-mentioned FDA-approved devices.

## Review

During the last 70 years, tremendous intervention and innovation have occurred in the field of percutaneous ASD closure using a transcatheter-based device [5]. Indications of ASD closure in adults include hemodynamically significant shunts leading to right heart enlargement, established paradoxical emboli, and platypnea-orthodeoxia syndrome (positional dyspnea and hypoxemia) [7,8].

Amplatzer Septal Occluder

The ASO is one of the most commonly used atrial septal occluders used these days. It has been approved by the FDA since 2001. The first FDA-approved study was conducted between September 1995 and May 1997 in Bratislava, Slovak Republic, and Boston, Massachusetts. In total, 30 patients (range = 2.9-62.4 years) underwent an attempt at ASD (median ASD diameter = 14mm) closure using the ASO on a 7-French-sized delivery catheter. The initial closure rate was 57%, as seen on transesophageal echocardiography (TEE) and angiography. The closure rate improved to 80% after 24 hours and two patients had a moderate residual shunt. Furthermore, the closure rate improved to 97% at one month which was the highest closure rate achieved by any device at that time. Many factors played a role in achieving a successful closure rate. The authors concluded that the unique design of the device, choosing patients and device size cautiously using TEE, and the ability to reposition the device before releasing the screw played a pivotal role in its success. A limitation of this clinical trial is that the application of the device was limited to small-sized ASDs, and larger devices have been available since then [9].

Jeffrey et al. published the results of the RESPECT trial, comparing medical therapy and PFO closure in reducing the risk of recurrent ischemic stroke. This multicenter randomized trial enrolled 980 patients between 18 and 60 years of age (median age = 45.9 years) with a history of ischemic stroke and randomly assigned them to either receive medical therapy or PFO closure. The median follow-up time was 5.9 years. The composite primary endpoint was either nonfatal death, recurrent nonfatal ischemic stroke, or early death after randomization. Recurrent ischemic stroke was classified as determined or undetermined using the ASCOD (atherosclerosis [A], small-vessel disease [S], cardiac pathology [C], other causes [O], dissection [D]) algorithm. In the intention-to-treat group, the rate of ischemic stroke was 0.58 events per 100 patient-years and 1.07 events per 100 patient-years in PFO closure and medical treatment groups, respectively (hazard ratio = 0.55; 95% confidence interval (CI) = 0.31 to 0.999; p = 0.046). Recurrent ischemic stroke of an undetermined cause was noted in 23 patients who underwent medical therapy and in 10 patients who underwent PFO closure using the ASO device. The authors, therefore, concluded that PFO closure was associated with a lower risk of recurrent ischemic stroke in patients with prior cryptogenic ischemic stroke than medical therapy alone during prolonged follow-up [10].

Closure of Atrial Septal Defects With the Amplatzer Septal Occluder Post-Approval Study (ASD PMS II) was a prospective, nonrandomized, multicentric study conducted in the United States between 2008 and 2012. The objective was to obtain survival data, safety, and effectiveness of the ASO in patients undergoing percutaneous closure of secundum ASDs with ASO at baseline, implant, one month, before discharge, and one month, one year, and two years post-implant. This was the largest prospective clinical trial that evaluated hemodynamic compromise in patients implanted with ASO. In total, 1,000 patients were enrolled. Inclusion criteria included secundum ASDs indicated for closure, willingness to sign an informed consent, and follow-up post-procedure. Age ranged from 0.3 years to 83.6 years (mean = 21 ± 22 years). The average static ASD diameter was 14.5 ± 6.2 mm, and the average stop-flow ASD diameter was 17.6 ± 6.2 mm. ASD closure was performed under echocardiographic imaging guidance 100% of the time, with TEE used in 58% of total patients, transthoracic echocardiography (TTE) in 6% of patients, and intracardiac echocardiography was used in 36% of patients. Successful closure of secundum ASD was observed in 97.9% of patients. A hemodynamic compromise was rare, only observed in 0.65% of total patients over two years. Cardiac erosion was only observed in 0.3% of patients, and only one such patient had a deficient aortic rim (aortic diameter <5 mm). The rate of device-related and device-delivery system-related adverse events was 6.5% of patients among 927 patients who completed the follow-up. The authors concluded that the rate of hemodynamic compromise was lower in patients who received an appropriately sized device. Device erosion must be considered in patients with hemodynamic compromise. The prevalence of deficient aortic rim was 11.5%. Limitations included a small sample size and inadequacy of septal rim echocardiographic data. Transcatheter closure of septum secundum ASD was safe and effective at two years [11].

A meta-analysis compared the incidence and risk of incident atrial fibrillation between the Amplatzer device (Amplatzer PFO occluder) and Gore devices (Cardioform and Helex septal occluders). All studies until July 2020 which mentioned the rate of atrial fibrillation in patients with cryptogenic stroke who underwent PFO closure using either of those devices were included. PubMed and Cochrane databases were utilized. The rate of incident atrial fibrillation in the Amplatzer group was 3.93% (30/763) versus 1.46% (11/751) in the respective medical group (relative risk = 2.57, p = 0.006) of all cases. Comparatively, the incidence of incident atrial fibrillation with the use of the GORE device was 6.57% (29/441) versus 0.44% (1/223) in the corresponding medical group (relative risk = 14.66, p = 0.008). The p-value for interaction between the two devices for the risk of atrial fibrillation was 0.10 [12].

Amplatzer Cribriform Occluder

The Amplatzer Cribriform Occluder is a septal occluder device developed by Abbott Laboratories intended for the closure of multifenestrated ASDs. Indications include cribriform ASDs in patients with evidence of right ventricular overload. It is a cribriform device with large and equal right and left discs with a connecting waist. In 2007, Mohammed et al. published data about the technical feasibility of this device when used to close fenestrated ASDs (F-ASDs). Their study’s inclusion criteria were the presence of F-ASDs diagnosed by TEE. Sixteen patients were included, with the majority being the pediatric population. Their sample size was small. Thirteen (81.2%) patients underwent successful transcatheter closure of secundum ASD. Overall, 76.9% of the patients ASDs were completely closed the next day. The remaining 4.3% of patients had full closure at six and 12 months. There was a statistically significant improvement in right ventricular diastolic pressure with mean pressure being reduced from 24.2 mmHg to 21.0 mmHg (p < 0.05). No strokes, conduction abnormalities, or deaths were observed. It was concluded that the Amplatzer Cribriform Occluder can be safely and successfully implanted in patients with F-ASDs [13].

Another study was published in 2016 which assessed the safety and efficacy of percutaneous, transcatheter closure of F-ASDs. A retrospective analysis was conducted to search for patients who have undergone such closure in the Ahmason/UCLA Adult Congenital Heart Disease Center Database. Eight such patients were identified who underwent transcatheter closure of F-ASDs and had associated pulmonary hypertension and/or left ventricular diastolic dysfunction or right ventricular dysfunction. Clinical follow-up revealed improvement in exercise tolerance and symptoms. Follow-up TTE data revealed patent fenestrations in four out of eight patients. No device-related complications, stroke, or infectious stigmata were observed. It was concluded that even partial ASD closure might be safe and effective in patients with F-ASDs and ventricular diastolic dysfunction and/or pulmonary arterial hypertension [14].

Gore Helex and Gore Cardioform Septal Occluder

In the REDUCE trial, PFO closure using the Gore Helex Septal Occluder (until late 2012), or the Gore Cardioform Septal Occluder (from late 2012), and antiplatelet therapy (PFO closure group) was compared with antiplatelet therapy alone. A total of 664 patients (median age = 45.2 years) were enrolled in this multicentric, randomized, prospective, controlled, open-label trial conducted in seven countries. Inclusion criteria included patients between the age of 18 and 59 years who had a cryptogenic ischemic stroke within 180 days of selection along with a diagnosed PFO with right-to-left shunt on TEE. The choice of antiplatelet depended on the local investigator. Either aspirin alone (50-100 mg daily), aspirin with dipyridamole (225-400 mg daily), or clopidogrel (75 mg once daily) were the choices. Primary endpoints included freedom from recurrent stroke over at least two years and incidence of new brain infarction detected on MRI. Secondary endpoints were successful PFO closure and rate of adverse events. PFO closure was performed in 93.7% of patients in the PFO with antiplatelet therapy group, and device retention was observed in 98.8% of patients. A different device was implanted in six patients. Successful occlusion was seen in 73.2% of patients immediately post-procedure and in 75.6% of patients at 12 months. Now looking at endpoints, recurrent stroke was seen in 1.4% of patients in the PFO closure group, and 5.4% of patients in the antiplatelet-only group (p = 0.002). Brain infarction was noted in 4.7% of patients in the PFO closure group and 10.7% of patients in the antiplatelet-only group (p = 0.002). The rate of overall serious adverse events was not statistically significant. The rate of atrial fibrillation or atrial flutter was much higher in patients who underwent device closure (6.6% vs. 0.4%, p < 0.001). We can conclude that PFO closure is associated with a statistically significant lower risk of recurrent brain infarction in patients with prior ischemic stroke within 180 days who undergo PFO closure compared to those who receive antiplatelet therapy alone [15]. The five-year follow-up data were consistent with the above results; however, data on new brain infarction were not collected [16].

Gore Cardioform ASD Occluder

The FDA approval for Gore Cardioform ASD Occluder (GCA) was received based on the results from the ASSURED clinical study. FDA approval was received in 2019. A total of 125 patients with secundum ASD were enrolled for fluoroscopy-guided closure of secundum ASD across 22 centers in the United States. Co-primary endpoints included closure success at six months and composite clinical success, defined as the following: successful deployment and retention of GCA (technical success), freedom from serious adverse events through 30 days post-procedure and freedom from device-related events through six months (safety success), and clinical residual defect status of occluded or clinically insignificant as determined at six months by the Echo Core Lab in patients with successful initial deployment and retention (closure success). The median age was 12.3 years within a range of 2.9 of 84.7 years. The median ASD diameter was 17.0 mm. A deficient retro-aortic rim was seen in 57% of patients. Additionally, 30% of patients had a median diameter of 18.0 mm with a deficient retro-aortic rim. Overall, 14.4% of patients had multiple fenestrations, while 4.8% of patients had an atrial septal aneurysm. Technical success was achieved in 96% of patients. Closure was attempted in 112 patients and was achieved in 100% of patients at six months. Serious adverse events were seen in 4.8% of patients at 30 days. Moreover, 4.8% of patients had new-onset clinically significant arrhythmia. Composite clinical success was seen in 90% of patients at six months. Overall, the GCA performed well, with a low rate of serious adverse events in 30 days, a low rate of new-onset arrhythmia, and a low rate of device events (2.4%) in six months, proving it to be safe and effective and subsequently getting FDA approved. There was no limitation in the age groups enrolled, which means that it can be used in a wide range of patients, regardless of deficient retro-aortic rim [17].

Comparison between transcatheter and surgical atrial septal defect closure

A meta-analysis was conducted to assess the safety, efficacy, and clinical utility of the ASO when compared with surgery for the procedural treatment of secundum ASDs [18]. In total, 596 patients were selected, of whom 442 patients underwent percutaneous closure while 154 patients underwent surgery. Safety was defined by the absence of mortality or major complications by a monitoring board. Major complications included cerebral embolism, cardiac perforation with tamponade, endocarditis, repeat operation, death, cardiac arrhythmias requiring permanent pacemaker placement or long-term antiarrhythmic medication, or device embolization requiring immediate surgical removal. The median age was 9.8 years for the ASO group and 4.1 years for the surgery group. Overall, 89.4% of the patients in the device group had a single ASD, while 80% of patients in the surgery group had a single ASD. Successful closure was defined by no, trivial (<1 mm color jet width), or small (1-2 mm color jet width) residual shunt on color Doppler echocardiography. The success rate was 95.7% for the ASO group and 100% for the surgical group (p = 0.006). The rate of adverse events was lower in the device group (7.4%) compared to the surgical group (24%) (p < 0.001). Patients in the transcatheter group also had a shorter length of stay in the hospital (1.0 ± 0.3 days) compared to patients who underwent surgical procedures for ASD closure (3.4 ± 1.2 days) (p < 0.001). Neither group had any mortality, and major complications were similar in both groups (1.6% in the device group vs. 5.4% in the surgery group, p: 0.03) [18,19].

Complications of transcatheter atrial septal defect closure

Various complications related to the device or the procedure itself have been described in the literature over the years. Several complications including femoral arteriovenous (AV) fistulas [20,21], device embolization [22], cardiac erosion [23], aortic incompetence [24], new-onset migraine [25], arrhythmias [26], thromboembolism [27], and left ventricular dysfunction [28] are well known. The rate of femoral AV fistulas remains low, only approaching 0.004-0.02% among a moderate sample size [20,21]. Device embolization is uncommon as well. Butera et al. reported less than 1% of cases of device embolization [22] among 1,013 patients with type II ASD in four years, similar to another study conducted in Korea [21]. Various factors that predispose to device embolization include larger defect size with floppy or deficient rims, thin atrial septal tissue, type of the device used, use of undersized device, and a change in the position of the device after its deployment. Excessive tension on the delivery cable or excessive wiggle maneuvering may increase the chances of embolization [29]. Cardiac erosion is a well-described but rare complication of ASD closure, and most life-threatening cases occur with the use of the ASO. Patients with oversized ASO, deficient retro-aortic rim, or a superior aortic rim appear to be at a higher risk of device erosion. This risk may be minimized by using an appropriately sized ASD occluder device and following such patients closely [23]. New-onset or worsening of aortic regurgitation (AR) can occur with percutaneous ASD or PFO closure. A study published in 2008 reported new-onset AR or worsening of AR in 9% of patients with an ASD and in 10% of patients with a PFO at 12 months. Cabau et al. reported the incidence of migraine headache attack with or without aura in 7% of patients post-ASD-PFO closure at a median 27-month follow-up. Data were gathered via a questionnaire (International Headache Society Criteria for migraine) in 185 (without a history of migraine) patients who underwent ASD-PFO closure. Migraine, mostly with aura, was associated with younger age (26 ± 16 vs. 39 ± 21 years; p = 0.02). These were patients who would have more likely undergone ASD closure (100% vs. 58%; p = 0.001) [24]. The occurrence of migraine headache with aura after the procedure was only predicted by ASD closure. These findings imply that factors beyond the composition of the device play a role in the development of migraine headache with aura in such cases. The incidence of atrial fibrillation was reported to be 3.9% [26]. The risk of recurrent neurologic thromboembolism may vary between 0% and 4.9% according to a retrospective analysis [27]. Ewert et al. measured left atrial pressure and mitral valve infilling pattern during total balloon occlusion of the ASD and after deflation in 18 patients over the age of 60 years. Early-to-late ventricular filling velocities of the mitral valve increased substantially (p = 0.02) with elevated atrial pressures (mean = 27 mmHg). This shows that an ASD may have a relieving effect on the left ventricle, which should be taken into consideration when planning an ASD closure in the elderly [28].

Devices not yet approved by the FDA

There are many CE (Conformité Européene) marked devices that are not approved by the FDA in the United States. Some of them include BioSTAR, CardioSEAL device, Solysafe Septal Occluder device, modified Rashkind PDA umbrella device, ATRIASEPT I-ASD device, ATRIASEPT II-ASD device, ULTRASEPT device, Sideris’ wireless devices, and PFM ASD-R device [30,31].

## Conclusions

Careful and litigious patient selection is the key to achieving successful results after PFO closure. Percutaneous closure of PFO and secundum ASDs is an appropriate choice for many patients. More data are needed on risk factors contributing to ischemic stroke in patients with PFO. Device sizing is key. No midline thoracotomy is required, and transcatheter closure is associated with shorter hospital stays. The success rate is proportional to surgical closure. Atrial arrhythmias are encountered more often in these patients, and further research is needed to minimize this risk.

## References

[1] Rojas CA, El-Sherief A, Medina HM, Chung JH, Choy G, Ghoshhajra BB, Abbara S (2010). Embryology and developmental defects of the interatrial septum. AJR Am J Roentgenol.

[2] Hoffman JI, Kaplan S (2002). The incidence of congenital heart disease. J Am Coll Cardiol.

[3] Menillo AM, Lee LS, Pearson-Shaver AL (2023). Atrial Septal Defect. https://www.ncbi.nlm.nih.gov/books/NBK535440/.

[4] Turner ME, Bouhout I, Petit CJ, Kalfa D (2022). Transcatheter closure of atrial and ventricular septal defects: JACC focus seminar. J Am Coll Cardiol.

[5] King TD, Mills NL (1974). Nonoperative closure of atrial septal defects. Surgery.

[6] (2023). A history of ASD closure. https://citoday.com/articles/2010-sept-oct/a-history-of-asd-closure#:~:text=Initial%20experimental%20attempts%20to%20surgically,closure%20in%20dogs%20in%201947.

[7] Baumgartner H, Bonhoeffer P, De Groot NM (2010). ESC Guidelines for the management of grown-up congenital heart disease (new version 2010). Eur Heart J.

[8] Liava'a M, Kalfa D (2018). Surgical closure of atrial septal defects. J Thorac Dis.

[9] Masura J, Gavora P, Formanek A, Hijazi ZM (1997). Transcatheter closure of secundum atrial septal defects using the new self-centering amplatzer septal occluder: initial human experience. Cathet Cardiovasc Diagn.

[10] Saver JL, Carroll JD, Thaler DE, Smalling RW, MacDonald LA, Marks DS, Tirschwell DL (2017). Long-term outcomes of patent foramen ovale closure or medical therapy after stroke. N Engl J Med.

[11] Turner DR, Owada CY, Sang CJ Jr, Khan M, Lim DS (2017). Closure of secundum atrial septal defects with the AMPLATZER Septal Occluder: a prospective, multicenter, post-approval study. Circ Cardiovasc Interv.

[12] Vukadinović D, Scheller B, Ukena C, Ewen S, Mahfoud F, Böhm M (2022). Device-related risk of atrial fibrillation after closure of patent foramen ovale: a systematic review and meta-analysis. Clin Res Cardiol.

[13] Numan M, El Sisi A, Tofeig M, Gendi S, Tohami T, El-Said HG (2008). Cribriform amplatzer device closure of fenestrated atrial septal defects: feasibility and technical aspects. Pediatr Cardiol.

[14] Abdelkarim A, Levi DS, Tran B, Ghobrial J, Aboulhosn J (2016). Fenestrated transcatheter ASD closure in adults with diastolic dysfunction and/or pulmonary hypertension: case series and review of the literature. Congenit Heart Dis.

[15] Søndergaard L, Kasner SE, Rhodes JF (2017). Patent foramen ovale closure or antiplatelet therapy for cryptogenic stroke. N Engl J Med.

[16] Kasner SE, Rhodes JF, Andersen G (2021). Five-year outcomes of PFO closure or antiplatelet therapy for cryptogenic stroke. N Engl J Med.

[17] Sommer RJ, Love BA, Paolillo JA, Gray RG, Goldstein BH, Morgan GJ, Gillespie MJ (2020). ASSURED clinical study: new GORE® CARDIOFORM ASD occluder for transcatheter closure of atrial septal defect. Catheter Cardiovasc Interv.

[18] Yang MC, Wu JR (2018). Recent review of transcatheter closure of atrial septal defect. Kaohsiung J Med Sci.

[19] Du ZD, Hijazi ZM, Kleinman CS, Silverman NH, Larntz K (2002). Comparison between transcatheter and surgical closure of secundum atrial septal defect in children and adults: results of a multicenter nonrandomized trial. J Am Coll Cardiol.

[20] Schwerzmann M, Windecker S, Wahl A (2004). Percutaneous closure of patent foramen ovale: impact of device design on safety and efficacy. Heart.

[21] Jung SY, Kim AY, Jung JW, Choi JY (2019). Procedural, early and long-term outcomes after percutaneous closure of atrial septal defect: comparison between large and very large atrial septal defect groups. Korean Circ J.

[22] Butera G, Romagnoli E, Carminati M (2008). Treatment of isolated secundum atrial septal defects: impact of age and defect morphology in 1,013 consecutive patients. Am Heart J.

[23] Amin Z, Hijazi ZM, Bass JL, Cheatham JP, Hellenbrand WE, Kleinman CS (2004). Erosion of Amplatzer septal occluder device after closure of secundum atrial septal defects: review of registry of complications and recommendations to minimize future risk. Catheter Cardiovasc Interv.

[24] Schoen SP, Boscheri A, Lange SA, Braun MU, Fuhrmann J, Kappert U, Strasser RH (2008). Incidence of aortic valve regurgitation and outcome after percutaneous closure of atrial septal defects and patent foramen ovale. Heart.

[25] Rodés-Cabau J, Mineau S, Marrero A (2008). Incidence, timing, and predictive factors of new-onset migraine headache attack after transcatheter closure of atrial septal defect or patent foramen ovale. Am J Cardiol.

[26] Staubach S, Steinberg DH, Zimmermann W, Wawra N, Wilson N, Wunderlich N, Sievert H (2009). New onset atrial fibrillation after patent foramen ovale closure. Catheter Cardiovasc Interv.

[27] Khairy P, O'Donnell CP, Landzberg MJ (2003). Transcatheter closure versus medical therapy of patent foramen ovale and presumed paradoxical thromboemboli: a systematic review. Ann Intern Med.

[28] Ewert P, Berger F, Nagdyman N, Kretschmar O, Dittrich S, Abdul-Khaliq H, Lange P (2001). Masked left ventricular restriction in elderly patients with atrial septal defects: a contraindication for closure?. Catheter Cardiovasc Interv.

[29] Batta A, Naganur S, Rajan A, Ary KA, Gawalkar A, Barwad P (2021). Retrieval and repositioning of an embolized atrial septal defect closure device using a gooseneck snare. Egypt Heart J.

[30] Rao PS (2020). Outcomes of device closure of atrial septal defects. Children (Basel).

[31] Alapati S, Rao PS (2012). Historical aspects of transcatheter occlusion of atrial septal defects. Atrial Septal Defect.

